# Immunogenicity and protective efficacy of recombinant *Clostridium difficile* flagellar protein FliC

**DOI:** 10.1038/emi.2016.8

**Published:** 2016-02-03

**Authors:** Chandrabali Ghose, Ioannis Eugenis, Xingmin Sun, Adrianne N Edwards, Shonna M McBride, David T Pride, Ciarán P Kelly, David D Ho

**Affiliations:** 1 Aaron Diamond AIDS Research Center, New York, NY 10075, USA; 2 Tufts University, Cummings School of Veterinary Medicine, North Grafton, MA 01536, USA; 3 Department of Microbiology and Immunology, Emory University School of Medicine, Atlanta, GA 30322, USA; 4 Departments of Pathology and Medicine, University of California, San Diego, La Jolla, CA 92093, USA; 5 Division of Gastroenterology, Beth Israel Deaconess Medical Center, Harvard Medical School, Boston, MA 02215, USA; 6 The Rockefeller University, New York, NY 10065, USA

**Keywords:** *C. difficile*, flagella, FliC, vaccine

## Abstract

*Clostridium difficile* is a Gram-positive bacillus and is the leading cause of toxin-mediated nosocomial diarrhea following antibiotic use. *C. difficile* flagella play a role in colonization, adherence, biofilm formation, and toxin production, which might contribute to the overall virulence of certain strains. Human and animal studies indicate that anti-flagella immune responses may play a role in protection against colonization by *C. difficile* and subsequent disease outcome. Here we report that recombinant *C. difficile* flagellin (FliC) is immunogenic and protective in a murine model of *C. difficile* infection (CDI) against a clinical *C. difficile* strain, UK1. Passive protection experiments using anti-FliC polyclonal serum in mice suggest this protection to be antibody-mediated. FliC immunization also was able to afford partial protection against CDI and death in hamsters following challenge with *C. difficile* 630Δ*erm*. Additionally, immunization against FliC does not have an adverse effect on the normal gut flora of vaccinated hamsters as evidenced by comparing the fecal microbiome of vaccinated and control hamsters. Therefore, the use of FliC as a vaccine candidate against CDI warrants further testing.

## Introduction

*Clostridium difficile* is the causative agent of nosocomial, antibiotic-associated infectious diarrhea. With the emergence of an epidemic strain of *C. difficile* (BI/NAP1/027) in the last decade, *C. difficile* infection (CDI) is now associated with a high rate of mortality.^1^ Symptomatic disease is caused by the actions of *C. difficile*'s two major toxins, toxin A (TcdA) and toxin B (TcdB), and the binary toxin (CDT) that is expressed by certain epidemic *C. difficile* strains.^2,3^ Antibodies against *C. difficile* are present in a majority of adults and older children through the transient exposure to *C. difficile*, possibly at infancy.^4,5^ No effective vaccine is commercially available although clinical evidence indicates that host immunity against *C. difficile* TcdA and TcdB in the form of toxin-neutralizing antibodies is protective.^6,7^ However, immune responses against toxins do not prevent colonization by *C. difficile*.^6,8,9^

As host immune responses to toxins play an important role in disease outcomes, recent studies have looked at the presence of adaptive immune responses to non-toxin virulence factors such as the S-layer proteins, cell wall proteins, and flagella in CDI patients.^10,11^ These studies show that antibody levels against surface proteins, such as flagella and cell wall protease 84 (Cwp84), were significantly higher in hospitalized patients who did not develop CDI than in a CDI patient group, suggesting a possible protective role of such immune responses.

Several studies have reported that flagellar proteins are highly immunogenic and that natural anti-flagella immune responses may play a role in protection against colonization.^12^ Adherence of non-flagellated strains of *C. difficile* to mouse cecum is 10-fold lower than for flagellated strains.^13^ Furthermore, flagellar protein-immunized mice showed reduced intestinal colonization by *C. difficile*.^10^ However, the role of flagella in the pathogenesis of *C. difficile* is complex, with recent studies providing new insights into the role of flagella not only in motility, but also in colonization, toxin gene expression, and biofilm formation.^14,15^

The *C. difficile* flagellum is composed of a membrane-bound basal body, a helicoidal filament and a hook. The 39 kDa flagellin, FliC, and the 56 kDa flagellar cap protein, FliD, are two components of the *C. difficile* flagellum.^16,17,18^ *fliC* and *fliD* are present on the F1 gene locus of the flagellar regulon, part of a cluster of late-stage flagellar genes.^19^ *fliC* and *fliD* are present and transcribed in all strains, either flagellated or non-flagellated, in *in vitro* culture. Thus, ‘non-flagellated' strains may possess cryptic *fliC* and *fliD* genes selectively expressed under certain *in vivo* conditions during host–pathogen interaction and may be essential for colonization.^20^ Conversely, recent analysis also shows that disease-causing epidemic 078 ribotype strains lack flagella.^3^

*C. difficile* FliC is well conserved in the N- and C-terminal regions, which are responsible for polymerization and secretion of FliC, whereas the central surface exposed region is variable.^17^ Although the central domain is divergent, polyclonal antibodies raised against FliC of one *C. difficile* strain react to FliC of other *C. difficile* strains, suggesting shared cross-reacting immunologic epitopes, in contrast to strains of *Clostridium sordellii* and *Bacillus subtilis*, which do not cross-react to *C. difficile* FliC.^16^ These observations suggest the presence of conserved cross-reactive elements within *C. difficile* FliC that is not typical for flagellar proteins in other species.

It has also been reported that *C. difficile* FliC is able to activate the nuclear factor kappa-light-chain-enhancer of activated B cells (NF-κB) pathway via signaling through Toll-like receptor-5 (TLR5).^21^ TLRs are a family of pattern recognition receptors that recognize structural components shared by bacteria, fungi, and viruses. TLRs, when bound to their ligands such as FliC, may trigger innate and adaptive immune responses, as well as facilitate the development of adaptive immunity through various mechanisms, such as the activation and maturation of dendritic cells and the expression of cytokines and other co-stimulatory proteins.^22^ The *Salmonella enterica* serovar *Typhimurium* FliC has previously been used with partial success as an adjuvant in experimental vaccines against influenza virus, *Vibrio cholerae*, and malaria.^23,24,25^ Recently, it has been reported that *S. Typhimurium* FliC-mediated stimulation of TLR5 in mice protects them from death during CDI by delaying *C. difficile* growth and toxin production in the gut.^26^ Whether *C. difficile* FliC-mediated activation of TLR5 can play a significant role in responses against *C. difficile* and its disease outcome is unknown.

We hypothesized that active immunization of a susceptible host using *C. difficile* flagellar components could provide both a preventive and a therapeutic vaccine strategy. In this study, we report that recombinant *C. difficile* flagellar protein FliC is immunogenic in mice, and immunization with FliC is protective in a murine model of CDI during challenge with a clinical *C. difficile* strain, UK1. Protection against CDI in mice following the passive transfer of anti-FliC polyclonal sera suggests that this protection is antibody-mediated. In addition, FliC was able to afford partial protection in a hamster model of CDI. These results suggest that a recombinant *C. difficile* FliC is an attractive candidate for further development and evaluation for active and passive immunization.

## Materials and Methods

### Bacterial strains

*Escherichia coli* DH5α and *E. coli* BL21DE3* (Life Technologies, Carlsbad, CA) were used for subcloning and recombinant protein purification, respectively. *C. difficile* UK1 (a 027/B1/NAP1 strain, kindly provided by Dale Gerding), a clinical strain isolated during a 2006 outbreak at Stoke-Mandeville hospital in the UK, was used for the bacterial challenge experiments in mice.^27^ *C. difficile* 630Δ*erm* (ribotype 012) was used for the bacterial challenge experiments in hamsters. *C. difficile* 630Δ*erm* is a spontaneous erythromycin-sensitive derivative of the parental reference strain 630 obtained by serial passaging in antibiotic-free media.^28^ It is well defined and widely used as a challenge strain in *C. difficile* hamster models of infection.^29,30^ *C. difficile* was propagated and spores were prepared as described previously.^31,32,33^

### Recombinant protein purification

The full-length protein sequence for FliC (NCBI AAD46086.1) was obtained from *C. difficile* strain VPI 10463 (ribotype 087; ATCC 43255). The receptor binding domains of TcdA (TcdARBD; NCBI M30307), and TcdB (TcdBRBD; NCBI P18177) were also identified and obtained from *C. difficile* strain VPI 10463.^21^ The corresponding nucleotides (*fliC*, *tcdARBD*, and *tcdBRBD*) were codon-optimized for expression in *E. coli*, synthesized and sequences were confirmed (Blue Heron Biotechnologies, Bothell, WA, USA). These nucleotides were cloned into the *Nde*I and *Bam*HI sites present on the multiple cloning site (MCS) of the pET19b bacterial expression vector (EMD Millipore, Billerica, MA, USA). The pET19b expression vector carries an amino-terminal (N-terminus) polyhistidine (His) tag containing six His residues followed by the MCS.

*E. coli* BL21DE3* competent cells were transformed with the pET19b expression vectors containing *fliC*, *tcdARBD*, or *tcdBRBD* inserts. The cultures were grown in Luria Bertani (LB) medium containing ampicillin (100 mg/mL) at 37 °C with aeration. Isopropyl-beta-D-thiogalactopyranoside (IPTG; 0.1 mM) was used to induce the expression of the inserted nucleotides (Sigma Aldrich, St. Louis, MO, USA). The expressed proteins were purified using Talon His-tag purification resin according to manufacturer's specifications (Clontech Laboratories Inc., Mountain View, CA, USA). Protein was detected by sodium dodecyl sulfate polyacrylamide gel electrophoresis (SDS-PAGE) and immunoblotting using commercial anti-His tag antibody (Life Technologies). The His-tag was not removed following purification, given the low immunogenicity of such tags.^34,35^ Endotoxin was removed by using Endotrap Blue columns according to manufacturer's specifications (Hyglos GmbH, Bernried, Germany). Endotoxin levels present in the purified recombinant proteins was measured by ToxinSensor Gel Clot Endotoxin Assay Kit (Genscript, Piscataway, NJ, USA).

### Immunization regimen

All animal work was approved by the Institutional Animal Care and Use Committee at the Rockefeller University, New York.

To study the immunogenicity and adjuvanticity of recombinant FliC, we immunized cohorts of five female, 8- to 10-week-old, C57/BL6 mice (Jackson Laboratories, Bar Harbor, ME, USA; Table 1). Mice were intraperitoneally (i.p.) immunized with 25 µg of FliC, adjuvanted with 1:1 by volume of Imject alum (alum) that contains an aqueous solution of aluminum hydroxide (40 mg/mL) and magnesium hydroxide (40 mg/mL) plus inactive stabilizers (Life Technologies). Cohorts of mice were also immunized with a total of 25 µg of TcdARBD and TcdBRBD, or a total of 25 µg of TcdARBD and TcdBRBD, adjuvanted with FliC. Cohorts of control mice were immunized with saline. All cohorts were immunized on days 0, 14, and 28. Blood samples were collected, and serum was processed and stored from mice on days 0 (naive serum), 14, 28, and 42, as previously described.^36^ Stool pellets from each mouse from all the cohorts were collected on days 3, 4, and 5 following challenge. All cohorts of mice were challenged two weeks post-last immunization as described below.

In the dose escalation study of recombinant FliC, four cohorts of 8–10 female, age-matched 8- to 10-week-old, C57BL/6 mice (Jackson Laboratories, Bar Harbor, ME, USA) were i.p. immunized on days 0, 14, and 28 (three immunizations) or on days 0, and 14 (two immunizations; Table 1). Cohorts of mice received 5 µg or 25 µg total of FliC adjuvanted with 1:1 by volume of alum. The cohort of control mice was immunized with saline and 1:1 volume of alum. Blood samples were collected, serum was processed, and stored from all the mice 24 h before challenge and two weeks post-challenge from the surviving mice. All cohorts of mice were challenged two weeks post-last immunization as described below.

To study the protective efficacy of the recombinant FliC immunization in the hamster model of CDI, 5- to 6-week-old (80–100 g) male Golden Syrian hamsters (*Mesocricetus auratus*) were obtained from Harlan and were housed in sterile individual ventilated cages. Hamsters were i.p. immunized on days 0, 14, and 28 (three immunizations; Table 1). Cohorts of hamsters (*n* = 7) received 20 µg or 100 µg total of FliC adjuvanted 1:1 volume of alum. A separate cohort of control hamsters (*n* = 7) was i.p. immunized with saline and 1:1 volume of alum. Blood was collected, serum was processed, and stored from hamsters one week before challenge and two weeks post-challenge from surviving hamsters. Stool pellets from each hamster from all the cohorts was collected one week before challenge All cohorts of hamsters were challenged three weeks after the last immunization as described below. These experiments were repeated twice with a total of 14 hamsters in each cohort.

### Measurement of serum antibody responses of mice and hamsters

To better standardize the enzyme-linked immunosorbent assay (ELISAs) for consistency, reproducibility, and accuracy for the detection of immune responses to FliC, TcdARBD, and TcdBRBD in mice and hamster serum, we completed a checkerboard dilution series with various concentrations of mouse and hamster serum.^37^ We coated plates with 10 ng/well, 20 ng/well, 50 ng/well, 100 ng/well, 200 ng/well, and 500 ng/well of commercially available purified TcdA, TcdB (List Biological Laboratories, Campbell, CA, USA) or recombinant FliC in 50 mM carbonate buffer, pH 9.6 as described elsewhere.^21^ Briefly, we blocked plates with PBS-1% bovine serum albumin (BSA; Sigma Aldrich). We diluted immune and naive sera 1:10, 1:50, 1:250, 1:500, 1:1000, 1:2000, 1:5000, and 1:1:10 000 in PBS containing 0.05% Tween 20 (PBS-T) and 0.1% BSA (Sigma Aldrich). We used a dilution of 1:500, 1:1000, and 1:2000 of goat anti-mouse immunoglobulin G (IgG) conjugated with horseradish peroxidase (HRP) and 1:2000, 1:4000, and 1:8000 of goat-anti-hamster IgG conjugated with HRP to detect bound antibodies (Southern Biotech, Birmingham, AL). We developed the plates with 2, 2'-azino-bis (3-ethylbenzthiazoline-6-sulfonic acid, ABTS; Sigma Aldrich) and 0.03% H_2_O_2_ (Sigma Aldrich) and determined optical density using a Vmax microplate reader (Molecular Devices Corp, Sunnyvale, CA, USA) at 405 nm kinetically for 5 min at 14-s intervals, as previously reported.^36^ We used a kinetic ELISA where data are expressed as change in milli-optical density units over time (OD/min).^38,39^

For the detection of antibody responses to FliC, TcdARBD, and TcdBRBD, we coated plates with 100 ng/well of purified TcdA, TcdB, or recombinant FliC in 50 mM carbonate buffer, pH 9.6. For the detection of anti-FliC, anti-TcdARBD and anti-TcdBRBD immune responses in serum, we diluted sera 1:1000 in PBS containing 0.05% Tween 20 (PBS-T) and 0.1% BSA. We used a dilution of 1:1000 of goat anti-mouse IgG conjugated with HRP and 1:8000 of goat-anti-hamster IgG conjugated with HRP to detect bound antibodies. We developed the plates as mentioned above and determined optical density via kinetic ELISA. Controls comprised of pooled serum from naive experimental cohorts. All samples were tested in technical duplicate.

### CDI model in mice

Mice were challenged via oral gavage two weeks after the last immunization. Mice were given orally administered antibiotic cocktail (kanamycin 40 mg/kg, gentamicin 3.5 mg/kg, colistin 4.2 mg/kg, metronidazole 21.5 mg/kg, and vancomycin 4.5 mg/kg) in drinking water for five days followed with i.p. administered clindamycin (10 mg/kg) 24 h before challenge. Mice were orally challenged with 10^6^ spores of strain *C. difficile* UK1 and monitored daily for 14 days for changes in weight, diarrhea, morbidity, moribundity and mortality.^32^ Mice were sacrificed when the following conditions were observed: rapid or progressive weight loss of greater than 25% of starting weight, a lack of responsiveness to manual stimulation, immobility, ruffled fur, hunched position, or signs of diarrhea, such as wet tail.

### Culture of *C. difficile* from mice fecal pellets

Fecal pellets were collected from each mouse on days 3, 4, and 5 post-challenge for the evaluation of *C. difficile* colonization. Freshly collected fecal pellets were weighed and stored in PBS at –80 °C.^40^ Spore counts of *C. difficile* were calculated by combining fecal samples of each mouse and plating serial dilutions on cycloserine-cefoxitin fructose agar plates with horse blood and taurocholate (CCFA-HT; Anaerobe Systems, Morgan Hill, CA, USA). Plates were incubated anaerobically for 48 h. Grayish-white colonies were manually counted.

### Passive transfer of polyclonal anti-FliC antibodies in mice

Ten female, 8- to 10-week-old, C57/BL6 mice were i.p. immunized three times every two weeks with 25 µg of FliC adjuvanted with alum. Hyperimmune serum was collected two weeks after the last immunization. Anti-FliC-specific serum IgG levels were measured by using a FliC-specific ELISA.

w?>Ten naive age-matched 8- to 10-week-old, C57BL/6 mice were given orally administered antibiotic cocktail (kanamycin 40 mg/kg, gentamicin 3.5 mg/kg, colistin 4.2 mg/kg, metronidazole 21.5 mg/kg, and vancomycin 4.5 mg/kg) in drinking water for 5 days followed with i.p. administered clindamycin (10 mg/kg) 24 h before challenge. At the time of clindamycin administration, 400 µL of FliC-specific hyperimmune serum was i.p. administered to five mice and 400 µL naive serum from unimmunized mice was given to the remaining five mice by i.p. administration. Twenty-four hours post-passive transfer of hyperimmune or naive serum, all 10 mice were challenged with 10^6^ UK1 spores, as described above. Blood was collected at the time of passive transfer (*t* = 0), day 1 (at time of challenge), day 3, day 7, and day 10, to study the level of circulating FliC-specific serum IgG in the blood. Following oral challenge, mice were monitored daily for 14 days as mentioned above.

### CDI model in hamsters

Hamsters were challenged three weeks after last immunization. All hamsters were dosed orogastrically with clindamycin (30 mg/kg) and infected five days later with 500 colony-forming units (CFU) of *C. difficile* strain 630Δ*erm*. Following oral challenge, hamsters were monitored every eight h for signs of disease progression for the duration of 14 days. Hamsters were sacrificed when the following conditions were observed: rapid or progressive weight loss of greater than 20%, a sign of recumbancy, such as leaning or having a hunched position, a lack of responsiveness to manual stimulation, or signs of diarrhea.

### Analysis of 16S rRNA fecal bacterial communities in hamsters

Fresh fecal samples were collected from hamsters one week prior to challenge and genomic DNA was prepared from each hamster using the Qiagen QIAamp DNA Stool Mini kit (Qiagen, Valencia, CA, USA). We amplified the bacterial 16S rRNA V1-V2 hypervariable region from the genomic DNA using the forward primer 8F (AGA GTT TGA TCC TGG CTC AG) fused with the Ion Torrent Adaptor A sequence and 1 of 23 unique 10 base pair (bp) barcodes, and reverse primer 357R (CTG CTG CCT YCC GTA) fused with the Ion Torrent Adaptor P1 from each fecal sample.^41^ Polymerase chain reactions (PCR) were performed using Platinum PCR SuperMix (Life Technologies) with the following cycling parameters: 94 °C for 10 min, followed by 30 cycles of 94 °C for 30 s, 53 °C for 30 s, 72 °C for 30 s, and a final elongation step of 72 °C for 10 min. Resulting amplicons were purified on a 2% agarose gel stained with SYBR Safe (Life Technologies) using the MinElute PCR Purification kit (Qiagen). Amplicons were further purified with Ampure beads (Beckman-Coulter, Brea, CA, USA), and molar equivalents were determined for each sample using a Bioanalyzer 2100 HS DNA kit (Agilent Technologies, Santa Clara, CA, USA). Samples were pooled into equimolar proportions and sequenced on 314 chips using an Ion Torrent PGM according to manufacturer's instructions (Life Technologies).^42^ Resulting sequence reads were removed from the analysis if they were <180 bp, had any barcode or primer errors, contained any ambiguous characters, or contained any stretch of >8 homopolymers. Sequences were assigned to their respective samples based on a 10-nucleotide barcode sequence, and were analyzed further using the Qiime pipeline.^43^ Briefly, representative Operational Taxonomic Units (OTU) from each set were chosen at a minimum sequence identity of 97% using UClust^44^ and aligned using PyNast^45^ against the Greengenes database.^46^ Multiple alignments then were used to create phylogenies using FastTree^47^, and taxonomy was assigned to each OTU using the Ribosomal Database Project (RDP) classifier.^48,49^ Principal coordinates analysis (PCoA) was performed based on Beta Diversity using weighted Unifrac distances.^50^

### Statistical analysis

For normally distributed data, we used an unpaired Student *t*-test analysis for comparison of means; for nonparametric data, we used the Mann–Whitney *U*-test. We performed statistical analyses using Microsoft Excel 2002 and plotted graphs using GraphPad Prism (GraphPad Software, San Diego, CA, USA). A *P*-value of less than 0.05 was considered to indicate statistical significance. Kaplan–Meier plots were used to analyze survival in the challenged mice, Mantel–Cox test for pairwise comparisons and Gehan Breslow Wilcoxon test was used to assess statistical significance between the cohorts.

## Results

### Cloning, expression and purification of recombinant *C. difficile* flagellar proteins FliC

**Figure 1:**
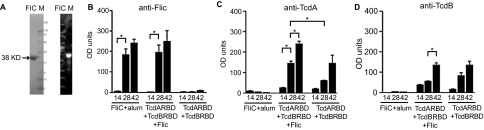
Immunogenicity and adjuvanticity of recombinant *C. difficile* flagellar protein FliC in mice. (**A**) 2 µg of purified recombinant *C. difficile* flagellar protein FliC was analyzed by SDS-PAGE on a gel stained with Coomassie blue (left panel). Whole cell lysate from uninduced and IPTG-induced BL21 (DE3)* cells were analyzed by Western blot using anti-His antibody (right panel). M, molecular weight markers; I, IPTG-induced cell lysate; U, uninduced cell lysate. Serum anti-FliC IgG (**B**), anti-TcdA IgG (**C**), and anti-TcdB IgG (**D**) responses in mice immunized on days 0, 14, and 28 in serum collected on days 14, 28, and 42. Cohorts of mice received 25 µg total of FliC adjuvanted with alum, unadjuvanted TcdARBD, and TcdBRBD or TcdARBD and TcdBRBD adjuvanted with FliC. Results were determined by kinetic ELISA and are reported as OD/min; the geometric mean plus standard error of the mean for each cohort is shown. * denotes statistical significance (*P* < 0.05), using an unpaired Student *t*-test analysis for comparison of means; for nonparametric data, we used the Mann–Whitney *U*-test.

We purified recombinant FliC (0.14 mg/mL) from *E. coli* using the pET19b expression plasmid. FliC was expressed as a 38 kDa protein (Figure 1A). Endotoxin levels were confirmed to be less than 5 endotoxin units/mL (EU/mL) in the purified protein samples. SDS-PAGE followed by Coomassie Blue staining confirmed the purity of recombinant FliC (Figure 1A, left panel). The identity of the His-tagged recombinant *C. difficile* flagellar protein FliC was confirmed by Western blot analysis using a anti-His monoclonal antibody (Figure 1A, right panel). TcdARBD and TcdBRBD were purified for an earlier study, and the purity of these proteins has been shown by SDS-PAGE elsewhere.^21^

### Immunogenicity and adjuvanticity of recombinant *C. difficile* flagellar protein FliC

For the detection of anti-FliC, anti-TcdARBD, and anti-TcdBRBD immune responses in serum by ELISA, 100 ng/well of recombinant FliC as capture antigen, mice or hamster serum dilutions of 1:1000, a 1:1000 dilution of goat anti-mouse IgG conjugated with HRP and a 1:8000 dilution of goat anti-hamster IgG conjugated with HRP provided consistent and accurate results (see Materials And Methods Section). A kinetic ELISA was used since this method produced true quantitative results requiring fewer multiple dilutions (data not shown).

Recombinant FliC was found to be immunogenic in mice, especially following two immunizations (*P* < 0.05, mean OD/min = 0.134 following two immunizations compared to mean OD/min = 0.05 following one immunization; Figure 1B). In mice that were immunized with FliC alone, a third immunization did not significantly improve the anti-FliC immune response in the serum of these mice compared to two immunizations IgG (*P* = 0.3429, mean OD/min = 0.134 following two immunizations compared to mean OD/min = 0.173 following three immunizations). This immune response was specific for FliC, as we do not see cross-reactive responses in mice immunized with TcdARBD and TcdBRBD (mean OD/min = 0.002, following three immunizations).^51^ Additionally, in the FliC-specific ELISA, cross-reactivity due to the presence of the low-immunogenic His-tag on the immunogen (TcdARBD and TcdBRDB) and the coating antigen (FliC) was not observed. Cohorts of mice that received FliC adjuvanted with alum had comparable levels of anti-FliC IgG levels to the cohort of mice that received TcdARBD and TcdBRBD in the presence of FliC following the second and the third immunizations (Figure 1B).

Next, we wanted to test the adjuvant properties of recombinant FliC. Following immunization with TcdARBD and TcdBRBD in the presence of FliC, mice were able to mount a stronger anti-TcdARBD IgG immune response (mean OD/min = 0.06) in serum compared to mice that received TcdARBD and TcdBRBD with no additional adjuvant help (mean OD/min = 0.02; Figure 1C). This significant enhancement of anti-TcdARBD IgG response was evident following the first boost (*P* < 0.05). This enhancement was not observed for anti-TcdBRBD IgG responses. Similar levels of anti-TcdBRBD IgG response were observed in the cohort that received TcdARBD and TcdBRBD adjuvanted with FliC and the cohort that received TcdARBD and TcdBRBD with no additional adjuvant help (*P* = NS; Figure 1D).

### Protective efficacy of recombinant *C. difficile* flagellar protein FliC and the receptor binding domains of TcdA and TcdB

**Figure 2:**
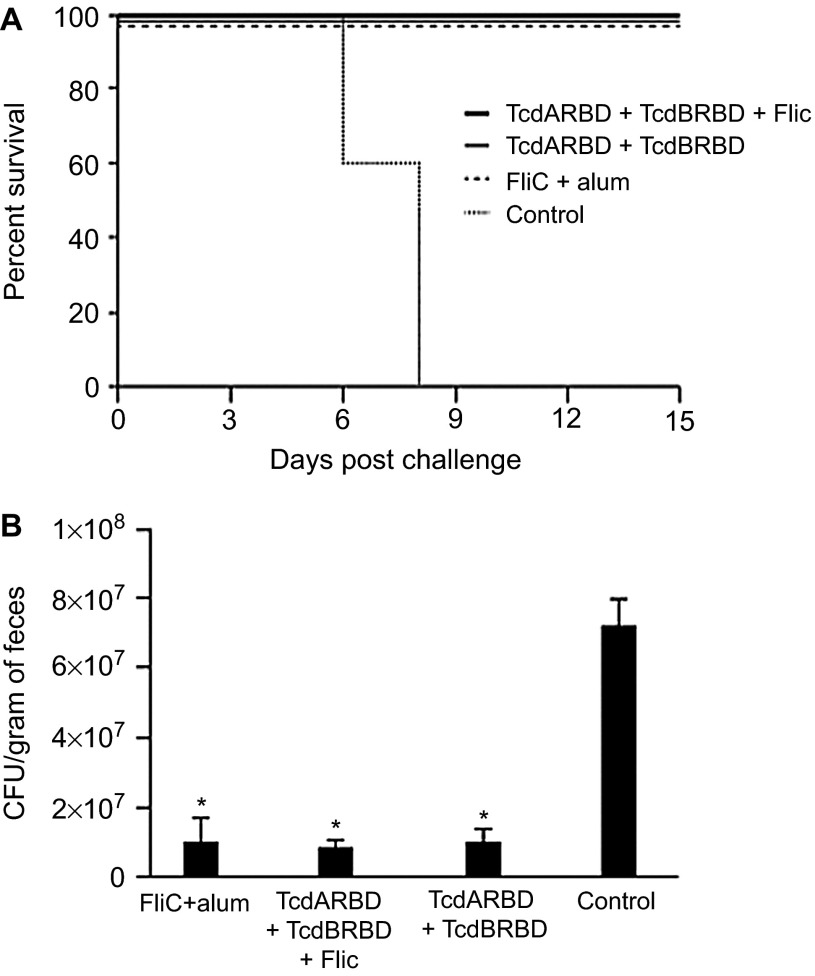
Survival and spore shedding in FliC-immunized mice following *C. difficile* UK1 challenge. (**A**) Survival in vaccinated C57BL/6 mice following orogastric challenge with 10^6^ CFU of *C. difficile* strain UK1. Cohorts of mice received 25 µg total of FliC adjuvanted with alum, unadjuvanted TcdARBD, and TcdBRBD or TcdARBD and TcdBRBD adjuvanted with FliC. Control mice were immunized with saline. Mice were immunized on day 0, 14, and 28 and challenged two weeks after the last immunization following antibiotic treatment. Mantel–Cox test for pairwise comparisons and Gehan Breslow Wilcoxon test was used to assess statistical significance between the cohorts. (**B**) *C. difficile* shedding in mouse fecal samples from immunized and control mice following orogastric challenge with 10^6^ CFU of *C. difficile* strain UK1. Results denote fecal shedding of *C. difficile* reported as CFU per gram (the geometric mean plus standard error of the mean) from fecal pellets. One fecal pellet was collected on days 3, 4, and 5 post-challenge from every mouse in each cohort containing five mice. * denotes statistical significance (*P* < 0.05), using an unpaired Student *t*-test analysis for comparison of means.

To test whether the recombinant FliC was able to induce a protective immune response, immunized mice from the previous experiments were challenged with *C. difficile* two weeks after the last immunization. The cohort of mice that was immunized with recombinant flagellar protein FliC adjuvanted with alum demonstrated 100% protection against CDI following challenge with a heterologous, clinically relevant *C. difficile* strain, UK1 (Figure 2A). Cohorts of mice immunized with TcdARBD and TcdBRBD alone or adjuvanted with FliC were also afforded 100% protection and remained completely disease-free until the end of the study when these mice were euthanized. In comparison, the control mice all succumbed to CDI by day 8 (*P* = 0.003). Hunched posture, ruffled fur, and signs of wet tail were observed in individual mice that were euthanized.

To test whether immunization with FliC would have an effect on the levels of spore shedding in *C. difficile*-challenged mice, we compared the levels of spores present in the in fecal pellets of immunized and control mice over a period of three days, starting from day three. Cohorts of mice immunized with TcdARBD and TcdBRBD alone or adjuvanted with FliC and the cohort of mice that was immunized with FliC adjuvanted with alum shed similar levels of *C. difficile* in their stool (*P* = NS; Figure 2B). All cohorts of immunized mice had significantly lower levels of *C. difficile* in their stool compared to control mice for up to day 5 post-challenge following which the control mice began to succumb to disease *(P* < 0.05; Figure 2B).

### Dose–response effect of recombinant *C. difficile* flagellar protein FliC on protective efficacy in mice

**Figure 3:**
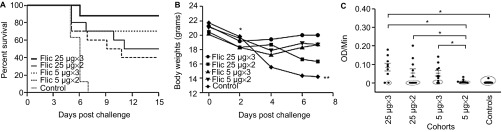
Anti-FliC IgG dose response and survival in FliC immunized mice challenged with *C. difficile* UK1. (**A**) Survival in vaccinated C57BL/6 mice following orogastric challenge with 10^6^ CFU of *C. difficile* UK1. Cohorts of mice received either 5 µg or 25 µg total of FliC adjuvanted with alum. Control mice were immunized with saline. Mice were immunized on day 0, 14, and 28 (three immunizations) or on days 0 and 14 (two immunizations) and challenged two weeks after the last immunization following antibiotic treatment. Mantel–Cox test for pairwise comparisons and Gehan Breslow Wilcoxon test was used to assess statistical significance between the cohorts. (**B**) Percent weight change of surviving mice compared to prechallenge (baseline weight), day 2, day 4, and day 7 following orogastric challenge with 10^6^ CFU *C. difficile* UK1. Results are reported as the geometric mean plus standard error of the mean for each cohort for each day. All mice in all cohorts are alive at day 2, all control mice succumb to CDI at day 7. * denotes statistical significance (*P* < 0.05), using an unpaired Student *t*-test analysis for comparison of means; when compared to the control cohort for each time point. (**C**) Anti-FliC IgG responses in serum of mice one day before challenge. Results were determined by kinetic ELISA and are reported as OD per minute; the geometric mean plus standard error of the mean for each cohort is shown. Data points circled denote mice that succumbed to challenge.) * denotes statistical significance (*P* < 0.05), using an unpaired Student *t*-test analysis for comparison of means; for nonparametric data, we used the Mann–Whitney *U*-test.

To further study the effect of dose–response and the number of immunizations on the protective efficacy of recombinant FliC, we immunized mice with a low or a high dose of FliC, either 5 µg or 25 µg, and varied the number of doses to either two or three i.p. immunizations, followed by heterologous challenge with *C. difficile* strain UK1 (Figure 3). The first symptoms of CDI presented on day 5 in four control mice, two mice immunized twice with 25 µg of FliC, and three mice each immunized with five µg of FliC either twice or three times (Figure 3A). The cohort of mice that received 25 µg of FliC and a total of three doses had the highest protective efficacy of 89%, with eight out of nine mice remaining disease-free following the challenge with *C. difficile* until the end of study when they were euthanized (*P* < 0.05, when compared to the control cohort). The cohort of mice that received 25 µg of FliC and a total of two doses had a protective efficacy of 50% (*P* < 0.05, when compared to the control cohort). Cohorts of mice that received 5 µg of FliC and three doses had a protective efficacy of 70% (*P* < 0.05, when compared to the control cohort). Cohorts of mice that received 5 µg of FliC twice had 40% protective efficacy (*P* = 0.01, when compared to the control cohort). Protective efficacy of the recombinant FliC vaccine also correlated with weight loss, with the cohort of mice receiving 25 µg of FliC and a total of three doses had the least weight loss due to diarrhea and colitis, as compared to the mice in the control cohort for days 4 and 6 (*P* < 0.005; Figure 3B). Mice that were euthanized that had greater than 20% weight loss, signs of diarrhea on their tail, and such symptoms were accompanied with a hunched posture and low levels of mobility. At day 7, the last surviving control mouse was weighed and euthanized, and changes in weight were no longer measured for the other surviving mice.

To correlate protection in mice with level of antibodies present in the blood, we looked for the presence of anti-FliC IgG in the serum of mice 24 h before challenge (Figure 3C). Mice that were immunized with the high 25 µg dose of FliC, either two (mean OD/min = 0.054) or three (mean OD/min = 0.083) times, as well as mice that were immunized three times with the low 5 µg dose of FliC (mean OD/min = 0.051), were able to mount a significantly stronger anti-FliC IgG response compared to the cohort that was immunized twice with the low 5 µg dose of FliC (mean OD/min = 0.005; *P* < 0.05). Additionally, mice in all cohorts that succumbed to challenge with *C. difficile* had lower levels of anti-FliC IgG in their serum 24 h before challenge than protected mice (mean OD/min = 0.001 vs. 0.081, *P* < 0.001; Figure 3C). Two weeks post-challenge, anti-FliC IgG was detected in the serum of all surviving mice. Anti-FliC IgG response was significantly higher only for the cohort of mice that received 25 µg of FliC two times, compared to anti-FliC IgG levels before challenge (*P* < 0.05; data not shown).

### Effect of passive transfer of mouse anti-FliC-specific polyclonal antibodies on the protective efficacy in mice

To assess the impact of FliC-specific antibodies on the protection against *C. difficile* challenge, hyperimmune serum was collected from mice immunized with recombinant FliC. The hyperimmune serum had an OD/min value of 0.345 using an anti-FliC IgG-specific ELISA. Based on our previous challenge experiments (Figure 3C), a measureable circulating anti-FliC IgG serum titer of greater than 0.0255 OD/min is required for 100% protection against *C. difficile* UK1. We transferred 400 µL of hyperimmune serum with an OD/min value of 0.345 to naive mice that were then challenged with *C. difficile* UK1 24 h post-infusion. Accounting for the total blood volume of an 8- to 10-week-old, C57/BL6 mouse to be two mL, a fivefold dilution of the infused hyperimmune serum would lead to a circulating anti-FliC IgG serum titer of 0.069 OD/min, which is still 2.7-fold greater than the protective titer needed for 100% protection.

**Figure 4:**
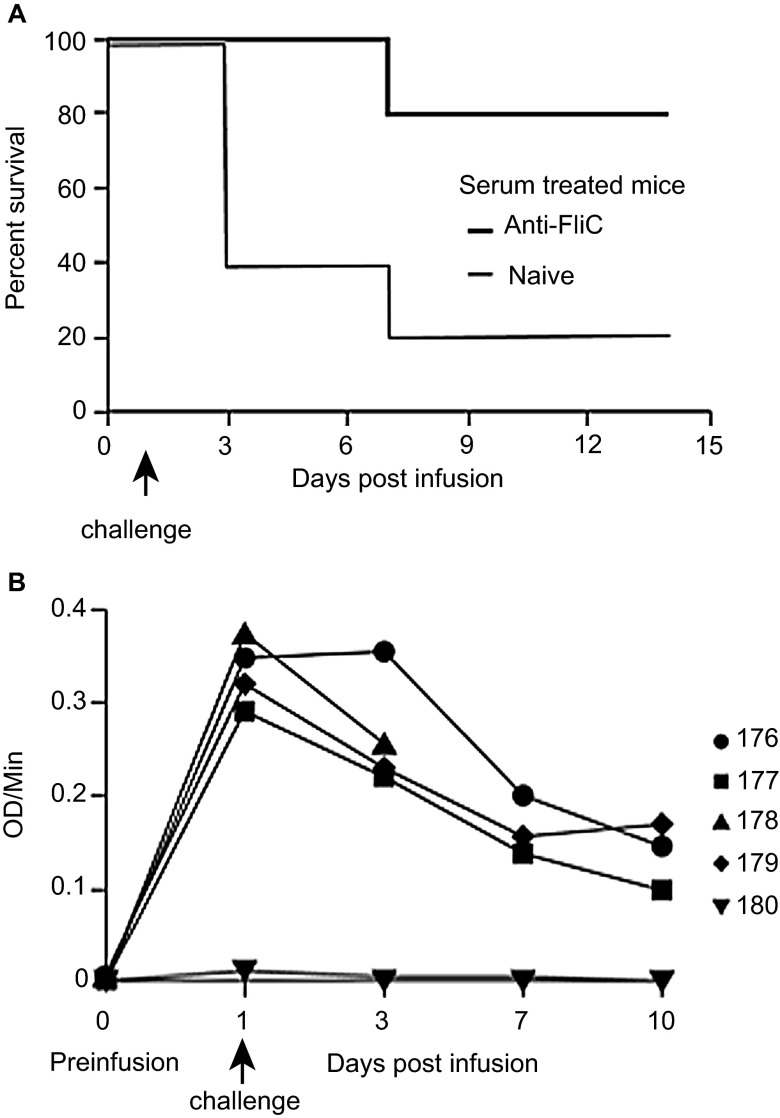
Protection of passively immunized C57BL/6 mice from challenge with *C. difficile* UK1. (**A**) Survival in passively immunized C57BL/6 mice following orogastric challenge with 10^6^ CFU of *C. difficile* UK1. Cohorts of mice received either 400 µl total of hyperimmune FliC serum or naïve serum. Antibiotic-treated mice were transfused on day 0 and challenged 24 h later. Mantel–Cox test for pairwise comparisons and Gehan Breslow Wilcoxon test was used to assess statistical significance between the cohorts. (**B**) Detection of circulating anti-FliC IgG on days 1, 3, 7, and 10 in serum of mice passively immunized with hyperimmune FliC serum. Results were determined by kinetic ELISA and are reported as OD per minute. Numbers denote individual mice. Mouse 178 succumbed to challenge on day 7, therefore no serum sample was collected for days 7 and 10.

Following *C. difficile* UK1 challenge, four out of five control mice that received 400 µL naive serum succumbed to *C. difficile* challenge between three and seven days post-challenge (Figure 4A). Signs of morbidity, such as hunched posture and ruffled fur were observed, along with signs of diarrhea and weight loss, and these mice were euthanized. Passive transfer of hyperimmune polyclonal anti-FliC serum was able to protect four out of five mice from infection following *C. difficile* challenge (*P* < 0.05 compared to the naive serum-treated mice). Passively administered anti-FliC antibody titers were detected in four out of five mice at the time of infection and until day 10 (Figure 4B).

### Protective efficacy of recombinant *C. difficile* flagellar protein FliC in the hamster model of CDI

**Figure 5:**
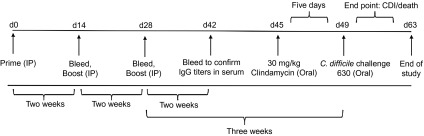
Experimental design of immunization and protective efficacy experiments in male Golden Syrian hamsters.

**Figure 6:**
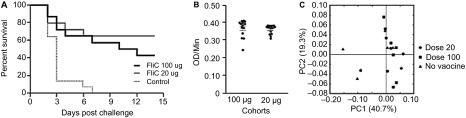
Survival in FliC-immunized golden Syrian hamsters following *C. difficile* 630Δ*erm* challenge. (**A**) Survival in control and vaccinated golden Syrian hamsters following orogastric challenge with 500 CFU of *C. difficile* 630Δ*erm*. Cohorts of hamsters received either 20 µg or 100 µg of FliC adjuvanted with alum. Control hamsters were immunized with saline. Mantel-Cox test for pairwise comparisons and Gehan Breslow Wilcoxon test was used to assess statistical significance between the cohorts. (**B**) Anti-FliC IgG responses in serum of vaccinated hamsters 1 week before challenge. Results were determined by kinetic ELISA and are reported as OD per minute; the geometric mean plus standard error of the mean for each cohort is shown. An unpaired Student *t*-test analysis was used for comparison of means. (**C**) PCoA of beta diversity present in the fecal bacterial communities in hamsters one week before challenge as measured by 16S rRNA. Animals that received no vaccine are represented by black triangles, 20 µg of vaccine are represented by circles, and 100 µg of vaccine are represented by squares.

Golden Syrian hamsters have been extensively used to study CDI due to their high level of susceptibility to *C. difficile* following the administration of antibiotics. To evaluate the protective efficacy of the recombinant FliC, hamsters were immunized and challenged with *C. difficile* 630Δ*erm* as shown in Figure 5. Based on our initial findings in the murine model (Figure 3A), cohorts were given three i.p. immunizations of either a low dose of 20 µg or a high dose of 100 µg of recombinant FliC adjuvanted with alum. The immunization dose of the low and high group reflect the dose adjustment due to the weight differences between mice and hamsters, which is approximately fourfold to fivefold. We observed that all animals in the control group that was immunized with saline and alum developed symptoms of CDI and were euthanized within 2–7 days after challenge with *C. difficile* 630Δ*erm* (Figure 6A). Hamsters were euthanized when signs of diarrhea were observed, such as wet tail, or had lost greater than 20% of their body weight. In the vaccinated cohorts, 8 out of 14 hamsters in the high dose group (43% survival) succumbed to CDI as compared to 5 out of 14 in the low dose group (64% survival). Mantel–Cox analysis revealed no statistically significant differences in the survival between the two vaccinated cohorts *(P =* 0.349) whereas the high dose and the low dose cohorts were significantly different from the control (*P* < 0.05). To test for correlations between the observed partial protection in hamsters and the level of circulating anti-FliC IgG present in the blood, we assessed the anti-FliC IgG in the serum of hamsters 1 week before challenge (Figure 6B). Irrespective of the dose the hamsters received, both cohorts had similar levels of anti-FliC IgG in their serum. We were also unable to detect any anti-toxin IgG in the serum of surviving hamsters two weeks post-challenge (data not shown).

We also characterized fecal bacterial communities in hamsters immunized with the recombinant FliC one week before challenge to determine whether immunization with FliC may have adversely affected their gut microbiota. Using PCoA, we observed that there were no distinguishing features of the fecal gut microbiota associated with the use of the recombinant vaccine when compared to control hamsters that did not receive the vaccine (Figure 6C).

## Discussion

Flagellum plays an important role in adhesion to mucus cells and colonization in several bacterial species such as *Helicobacter pylori*, *V. cholerae*, and *Pseudomonas aeruginosa*.^52^ Flagella-mediated motility, adherence and biofilm formation are important virulence factors that contribute to the overall fitness of pathogens, especially gastrointestinal pathogens. For *C. difficile*, the role of flagella in motility, adherence, and biofilm formation is strain-specific. For example, *C. difficile* strain R20291, a PCR ribotype 027 (B1/NAP1) hypervirulent strain isolated during an outbreak in the UK, is found to be monotrichously flagellated, with flagellum playing a role in colonization, adherence, and higher biofilm formation *in vitro*.^15,53^ This is in contrast to the widely used clinical strain *C. difficile* 630Δ*erm* which has peritrichous flagella, adhere less effectively and form a smaller biofilm *in vitro*.^28^ In this study, *C. difficile* strain 630Δ*erm* was able to cause CDI in hamsters starting at 48 h.

Given the importance of flagella in the virulence of *C. difficile*, active immunization of a susceptible host using *C. difficile* flagellar components in addition to TcdARBD and TcdBRBD could provide both a preventive and a therapeutic vaccine strategy by reducing or preventing bacterial colonization as well as preventing toxin-induced disease symptoms. Here we also report that mice immunized with TcdARBD and TcdBRBD adjuvanted with FliC had significantly higher anti-FliC IgG responses in the serum (Figure 1B and 1C) than the cohorts that received FliC adjuvanted with alum. The use of a single antigen without the aid of an additional adjuvant such as alum makes FliC an attractive vaccine candidate.^21,54^ For certain bacterial pathogens, such as *H. pylori*, persistence in their human host is possible by immune evasion. One such mechanism employed by *H. pylori* is to have flagellum that are less proinflammatory and do not activate TLR5.^55^ Although *C. difficile* FliC does activate TLR5 and has adjuvant properties associated with its TLR5 activation, this activation is at a much lower level than that observed with *S. Typhimurium* FliC, and immune evasion may be a reason for this.^21^ In this study, FliC does induce an adaptive immune response in mice and hamsters and is found to have adjuvant properties in the context of TcdARBD, but not with TcdBRBD. It is possible that the anti-TcdB IgG response is maximally adjuvanted with the co-administered TcdARBD, as it has been reported previously that TcdA has innate adjuvant properties.^56^ Whether *C. difficile* FliC-mediated activation of TLR5 plays a significant role in inducing innate immune responses against *C. difficile* was not addressed in our current study.

Bacterial challenge experiments in mice are restricted to a few *C. difficile* strains that can consistently infect and cause CDI in mice. In most cases, the strain VPI 10463, which expresses high levels of toxin A and B production, has low sporulation rates, and does not cause colitis in humans, has been used as the challenge strain.^57^ To more closely mimic the human disease, we have used a hypervirulent clinical strain, *C. difficile* strain, UK1 (027/B1/NAP1 strain) in our mice challenge model. The induced anti-FliC IgG response was protective in 88–100% of the mice following heterologous challenge with *C. difficile* UK1 in two separate challenge experiments in mice. FliC-immunized mice also shed fewer *C. difficile* spores in their stool between days 3 and 5 post-challenge. Although *C. difficile* spores are shed in the feces of mice for up to three weeks post-challenge, *C. difficile* spores rapidly transit through the gastrointestinal tract of mice and can be found in the feces of mice as early as four days, with the numbers significantly waning between days 7 and 10. In our current study, we analyzed whether vaccination with recombinant FliC or with the RBDs of toxins could lead to a difference in the level of shedding of *C. difficile* spores in these cohorts. Given that the control animals succumbed to CDI from day 6 post-challenge, for comparison between cohorts we restricted our study to the initial days post-challenge. Whether vaccination with a recombinant FliC vaccine prevents asymptomatic carriage of *C. difficile* following exposure needs to be further studied.

Protection from CDI and death in immunized mice correlated with a dose–response and the number of immunizations received, as well as the antibody titer levels before challenge. The mice that succumbed to challenge had lower levels of anti-FliC IgG response compared to those that survived. This phenomenon was observed across all cohorts of mice, suggesting that a strong anti-FliC IgG response is a correlate of protection in these immunized mice. Transfer of passive anti-FliC immunity in the form of hyperimmune polyclonal anti-FliC antibodies mediated protection against *C. difficile* challenge in four out of five mice, further underscoring the protective role of an adaptive immune response against *C. difficile* FliC. The mechanism by which parenterally induced IgG reaches the mucosal surface of the gut where *C. difficile* is present is unclear. One hypothesis is that FcRn receptors present in the epithelial cells of the gut are able to transport IgG across the intestinal epithelium or that small amounts are synthesized locally.^58,59^ A new model of protection against CDI in the gut lumen by systemic antibodies suggests that the transport of antibodies across the gut wall is not solely Fc-dependent but is due in part to the paracellular transport of systemic antibodies following toxin-mediate damage to the gut epithelium.^60^

Irrespective of the mechanism of protection and the site where circulating serum antibodies neutralize *C. difficile*, clinical studies and human studies continue to confirm the importance of circulating serum IgG in the protection against CDI.^6,61,62^ The importance of a mucosally induced IgA response in the protection from CDI remains unclear, and recent studies indicate that systemic IgG responses may be more important in determining clinical outcomes in CDI.^5,6,63^ Nevertheless, it would be beneficial for a parenteral vaccine to induce both a systemic and a mucosal immune response. We have previously demonstrated that a mucosal adjuvant such as a non-toxic mutant of *E. coli* heat labile enterotoxin, LT (r192g) is needed to induce a potent mucosal immune response in the form of IgA present in the stool of toxin-immunized mice.^21,36^ Further studies need to be performed to delineate the mechanism of protection of anti-FliC-induced IgA against CDI using mucosal adjuvants.

We confirmed our findings in mice with protective efficacy experiments in two separate challenge experiments in hamsters using strain *C. difficile* 630Δ*erm*. *C. difficile* 630Δ*erm* is a well-characterized clinical strain that consistently causes CDI in hamsters.^64^ Golden Syrian hamsters express TLR5 receptors (NCBI GeneID: 101829185) making this an appropriate model to confirm our previous results. In the hamster challenge experiments, we tested two doses based on our initial mouse experiments in which 5 µg or 25 µg of FliC given three times afforded high levels of protection. However, both cohorts of hamsters, given either a high or a low dose, had comparable levels of anti-FliC antibodies 1 week before challenge and were afforded partial protection against *C. difficile* 630Δ*erm* challenge. The observed level of protective efficacy of recombinant FliC in hamsters is comparable to other *C. difficile* surface antigens that have been tested in hamsters as vaccine candidates such as Cwp84 and the S-layer protein, SlpA (33% and 66% protective efficacy, respectively).^65,66^

Flagellin is highly variable across species. For example: *Bacillus sp*. C-125 flagellin is 31 kDa, *E. coli* flagellin is 51 kDa, and *B. subtilis* flagellin is 32 kDa. The smaller-sized flagellin lack an outer molecular domain present in higher molecular weight flagellin. Although highly variable, bacterial flagella share common epitopes due to their ability to globally activate TLR5. Anti-flagellin antibodies have been shown to be cross-reactive across different bacterial species due to these commonly shared epitopes and dysbiosis of the gut microflora may render the host more susceptible to pathogens such as *C. difficile*.^67^ We show here that using a *C. difficile* flagellin vaccination strategy does not have a significant impact on the membership of the fecal microbiome in hamsters and likely plays no role in inhibiting colonization of the indigenous bacterial communities of the hamster. These results suggest that any effects of the recombinant FliC vaccine were not mediated through changes in the bacterial membership of the gut microbiome. Moreover, patients who have CDI are able to mount an anti-FliC immune response but at the resolution of CDI, are still able to have a normal, functional gut thus showing no long-term side-effects of a natural anti-FliC immune response.^68^

Several studies suggest that toxin production in *C. difficile* is modulated positively, by genes from the flagellar (F)3 regulon, and negatively, by genes from the F1 region.^3,14^ Evidence also suggests that toxin expression is directly influenced by sigma factor D (SigD), the flagellar-specific sigma factor.^69^ It has been reported that inactivation of *fliC*, present in the F1 region of the flagellar regulon, leads to the overproduction of toxins in culture supernatants.^14^ To address whether vaccination of mice by the flagellar protein FliC would have an adverse effect on immunized mice leading to the production or even overproduction of toxins following challenge, we looked for anti-toxin IgG immune responses in mice that survived challenge. We were unable to detect any anti-toxin IgG in the serum of surviving mice 2 weeks post-challenge (data not shown). Additionally, mice that received multiple, higher doses of FliC had the highest survival rate, thus proving that there was no enhancement of infection due to toxin production from FliC vaccination.

The virulence of *C. difficile* is due to complex multifactorial interplay between several known virulence factors including the toxins and the flagellar proteins. TcdA and TcdB are essential for symptomatic disease, whereas the flagellar proteins are involved in motility, biofilm formation and toxin production.^14,70^ A vaccine strategy that targets single or multiple factors needed for colonization, persistence and toxin production presents a significant advantage over other vaccines currently in development. Our strategy of targeting the flagellar components, especially FliC, may have the capacity to counteract bacterial attachment and persistence and prevent symptomatic disease, and therefore, warrants further testing as a potential vaccine candidate. Further studies are currently ongoing to delineate the mechanism of protection of anti-FliC-induced antibodies against CDI.

## Figures and Tables

**Table 1:** Mice and hamster study design

Study	Immunization cohort	Immunization dose	Intraperitoneal immunization (i.p.) schedule	Number of animals	Orogastric challenge
Immunogenicity and adjuvanticity study in mice	FliC	25 μg FliC + 1:1 volume of Alum	Three immunizations on days 0, 14, 28	5	10^6^ spores of strain *C. difficile* UK1 on day 42
	TcdARBD + TcdBRBD	25 μg TcdARBD + TcdBRBD	Three immunizations on days 0, 14, 28	5	10^6^ spores of strain *C. difficile* UK1 on day 42
	TcdARBD + TcdBRBD + FliC	25 μg TcdARBD + TcdBRBD + FliC	Three immunizations on days 0, 14, 28	5	10^6^ spores of strain *C. difficile* UK1 on day 42
	Control	Saline + 1:1 volume of Alum	Three immunizations on days 0, 14, 28	5	10^6^ spores of strain *C. difficile* UK1 on day 42
Dose escalation study in mice	FliC 25 μg × 3	25 μg FliC + 1:1 volume of Alum	Three immunizations on days 0, 14, 28	9	10^6^ spores of strain *C. difficile* UK1 on day 42
	FliC 25 μg × 2	25 μg FliC + 1:1 volume of Alum	Two immunizations on days 0, 14	10	10^6^ spores of strain *C. difficile* UK1 on day 28
	FliC 5 μg × 3	5 μg FliC + 1:1 volume of Alum	Three immunizations on days 0, 14, 28	10	10^6^ spores of strain *C. difficile* UK1 on day 42
	FliC 5 μg × 2	5 μg FliC + 1:1 volume of Alum	Two immunizations on days 0, 14	10	10^6^ spores of strain *C. difficile* UK1 on day 28
	Control	Saline + 1:1 volume of Alum	Three immunizations on days 0, 14, 28	8	10^6^ spores of strain *C. difficile* UK1 on day 42
Protective efficacy study in hamsters	FliC 100 μg × 3	100 μg FliC + 1:1 volume of Alum	Three immunizations on days 0, 14, 28	14 (7 + 7)	500 cfu of *C. difficile* strain 630Δ*erm* on day 49
	FliC 20 μg × 3	20 μg FliC + 1:1 volume of Alum	Three immunizations on days 0, 14, 28	14 (7 + 7)	500 cfu of *C. difficile* strain 630Δ*erm* on day 49
	Control	Saline + 1:1 volume of Alum	Three immunizations on days 0, 14, 28	14 (7 + 7)	500 cfu of *C. difficile* strain 630Δ*erm* on day 49
